# Supplementary material for: Influence of light availability and soil productivity on insect herbivory on bilberry (Vaccinium myrtillus L.) leaves following mammalian herbivory
Source: PLoS One. 2020 Mar 27;15(3):e0230509. doi: 10.1371/journal.pone.0230509 (PMC7100976; doi:10.1371/journal.pone.0230509)
Supplement: S1 File — (PDF) [file pone.0230509.s001.pdf]

Supporting information in file 'S1 Supporting Information.pdf' for

Influence of light availability and soil productivity on insect herbivory on bilberry (*Vaccinium myrtillus* L.) leaves following mammalian herbivory

Marcel Schrijvers-Gonlag[1*], Christina Skarpe[1], Harry Peter Andreassen[1†]

[1] Campus Evenstad, Faculty of Applied Ecology, Agricultural Sciences and Biotechnology, Inland Norway University of Applied Sciences, Koppang, Norway

[*] Corresponding author, email: marcel.schrijversgonlag@inn.no

[†] deceased 21 May 2019

In this document:

- **Some statistics for the variables insect herbivory and previous mammalian herbivory and the variable shade**
- **The soil variables phosphorus (P) and nitrogen (N): some statistics and the PCA**
- **Insect herbivory versus mammalian herbivory on bilberry (figure)**
- **Ecological range for soil productivity of bilberry**
- **Temperature & precipitation in the study area within the study period**

Some statistics for the variables insect herbivory and previous mammalian herbivory and the variable shade

Mean insect herbivory was 2.0 % (range = 0 - 32 %, SE = 0.15 %). Edge herbivory (mean = 1.8 %, range = 0 - 30 %, SE = 0.14 %) was more frequent than hole herbivory (mean = 0.2 %, range = 0 - 5 %, SE = 0.03 %): in 436 quadrats (96 %) edge herbivory was present and in 272 quadrats (60 %) hole herbivory was present. In 438 quadrats (96 %) insect herbivory was present. Previous mammalian herbivory was present in 394 quadrats (87 %). Mean previous mammalian herbivory was 9.1 % (range = 0 - 80 %, SE = 0.67 %). In many quadrats we observed little herbivory: in 151 quadrats (33 %) previous mammalian herbivory was 1 % or less; insect herbivory was in almost half of all quadrats (n = 221, 49 %) 1 % or less (figure below). Many quadrats were at exposed and half-open locations (less than 20 % shade: n = 193, 42 %, shade between 20 and 80 %: n = 181, 40 %). Less than 20 % of all quadrats were in shady conditions (more than 80 % shade: n = 81, 18 %).

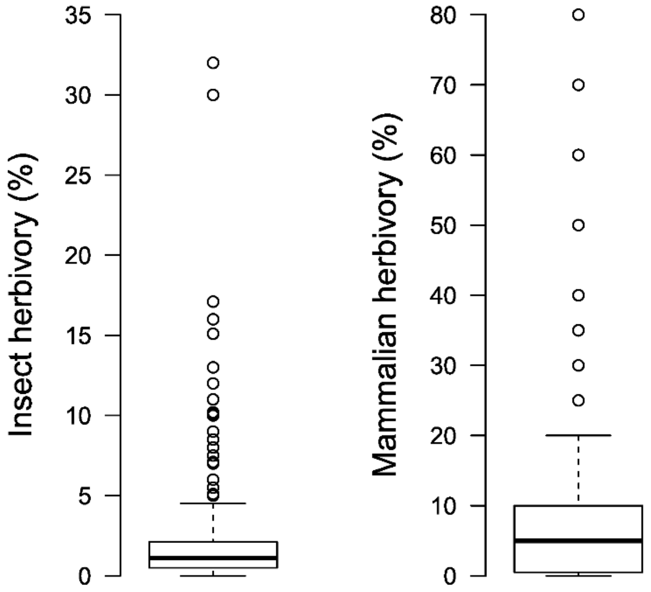

Insect and mammalian herbivory on bilberry. Mammalian herbivory = previous mammalian herbivory (see main text). The bottom and top of the box indicate the first and third quartiles. Bold horizontal lines within each box indicate median values. The plot whiskers extend to the most extreme data point which is no more than 1.5 times the interquartile range away from the box; more extreme data points are indicated with small circles. n = 455.

The soil variables phosphorus (P) and nitrogen (N): some statistics and the PCA

Correlation between ammonium lactate extractable phosphorus (P) and total nitrogen (N) was low (ρ = 0.181; as plotting P against N indicated that some values of N seemed to be small outliers, Spearman rank correlation coefficient was used). P and N had mean values and standard errors that differed by a factor 7 and 5, respectively (P: mean = 0.167 mg g -1, SE = 0.0035 mg g -1; N: mean = 1.23 % (dry matter), SE = 0.017 % (dry matter)). Consequently, a high difference in variance was present between P and N. Therefore we standardized the values for P and N before the PCA. The first and second principal component (PC1 and PC2) explained 55 % and 45 % of the total variation, respectively. In negative direction PC1 was equally loaded with P and N: eigenvector values were -0.742 for both variables. PC2 was positively loaded with P and negatively with N: eigenvector values were 0.671 and -0.671, respectively. We only used PC1 in our further analyses; PC1 is used as a composite covariate for soil productivity.

Insect herbivory versus previous mammalian herbivory on bilberry (figure)

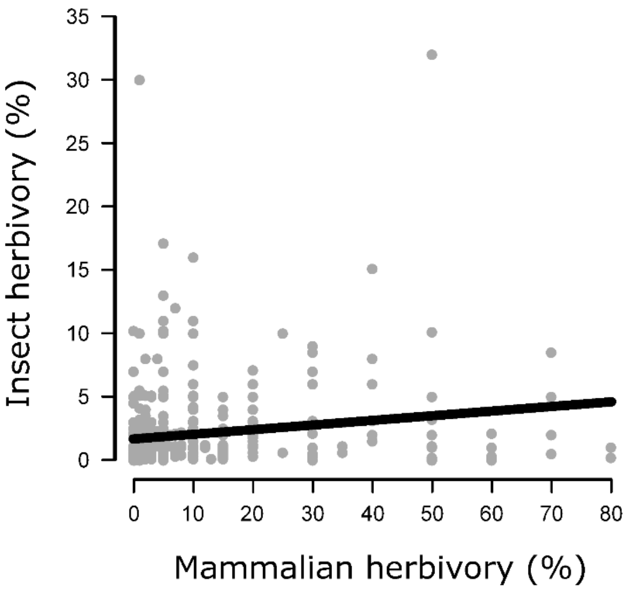

Insect herbivory versus mammalian herbivory on bilberry.

Mammalian herbivory = previous mammalian herbivory (see main text). Herbivory in this figure is independent of the variables shade condition and soil productivity. *P*-value (ANOVA; $F_{1,453}$ = 12.46) for the linear regression (regression line in black) = 0.00046, adjusted R-squared = 0.02, slope = 0.04, n = 455.

Ecological range for soil productivity of bilberry

Our observations may not cover the full ecological soil productivity range of bilberry (nitrogen and phosphorus levels showed little variation, see previous page), although bilberry cover in our data set ranged from 0.1 % to 94 % (first quantile = 7 %, median = 23 %, third quantile = 45 %, mean = 28 %, SE = 1.1 %) suggesting that both less favourable and highly prefered locations, and many in between, are included in our data set. This may correlate with a fairly broad soil productivity range, indicating that our observations may cover, or are close to, the full ecological soil productivity range of bilberry.

Temperature & precipitation in the study area within the study period

In the study area, 2014 was the warmest year since 1900, with more precipitation than in 2013 and 2015. In 2015 the mean temperature in May-August (the main growing season for bilberry) was almost 2 °C lower than in 2013 and 2014, and 0.5 °C lower than the long-term average for these months.

Source: Norwegian Meteorological Institute. Weather and climate data Norway. Available from: https://www.met.no; https://www.yr.no/sted/Norge/Østlandet/klima.html; http://eklima.met.no Accessed 9 January 2019.
