# Supplementary material for: Influence of light availability and soil productivity on insect herbivory on bilberry (Vaccinium myrtillus L.) leaves following mammalian herbivory
Source: PLoS One. 2020 Mar 27;15(3):e0230509. doi: 10.1371/journal.pone.0230509 (PMC7100976; doi:10.1371/journal.pone.0230509)
Supplement: S1 Table — Estimated marginal mean values for insect herbivory, their standard error, degrees of freedom and 95% confidence intervals are presented for each level of the variable shade in the shade model. Note that the model used a logit link function (the estimates are on a logit-scale, not the response scale) and that the response variable was transformed prior to analyses (see text in the manuscript). Therefore, also the back-transformed estimated marginal means (back-transformed from both logit transformation and response variable transformation) are presented (thus, these values are on the response scale). To make differences visible, three digits are given for the back-transformed estimated marginal means. Results are averaged over the levels of the variable year. Number of observations: 455. (PDF) [file pone.0230509.s002.pdf]

Table 1. Insect herbivory: estimated marginal means (EMMs) per shade level in the shade model. Estimated marginal mean values for insect herbivory, their standard error, degrees of freedom and 95 % confidence intervals are presented for each level of the variable shade in the shade model. Note that the model used a logit link function (the estimates are on a logit-scale, not the response scale) and that the response variable was transformed prior to analyses (see text in the manuscript). Therefore, also the back-transformed estimated marginal means (back-transformed from both logit transformation and response variable transformation) are presented (thus, these values are on the response scale). To make differences visible, three digits are given for the back-transformed estimated marginal means. Results are averaged over the levels of the variable year. Number of observations: 455.

**shade level**	**emmean**	**SE**	**df**	**lCI**	**uCI**	**BE**
Shade < 20 %	-4.01	0.07	448	-4.15	-3.86	0.017
Shade between 20 - 80 %	-3.77	0.07	448	-3.91	-3.63	0.021
Shade > 80 %	-3.68	0.10	448	-3.87	-3.49	0.024

emmean = estimated marginal mean; SE = standard error; df = degrees of freedom;
lCI = lower 95 % confidence interval; uCI = upper 95 % confidence interval; BE = back-transformed estimates.
