# Supplementary material for: Influence of light availability and soil productivity on insect herbivory on bilberry (Vaccinium myrtillus L.) leaves following mammalian herbivory
Source: PLoS One. 2020 Mar 27;15(3):e0230509. doi: 10.1371/journal.pone.0230509 (PMC7100976; doi:10.1371/journal.pone.0230509)
Supplement: S2 Table — Estimated marginal mean values for insect herbivory, their standard error, degrees of freedom and 95% confidence intervals are presented for each level of the variable shade in a linear model with and without year as a fixed effect. In the first model, results are averaged over the levels of the variable year. Similar information is presented for the contrast estimates; in addition, their P values based on Tukey's HSD test are given. Number of observations: 455. (PDF) [file pone.0230509.s003.pdf]

Table 2. Insect herbivory: estimated marginal means (EMMs) per shade level in a linear model and the contrast estimates with Tukey's HSD test values. Estimated marginal mean values for insect herbivory, their standard error, degrees of freedom and 95 % confidence intervals are presented for each level of the variable shade in a linear model with and without year as a fixed effect. In the first model, results are averaged over the levels of the variable year. Similar information is presented for the contrast estimates; in addition, their *P* values based on Tukey's HSD test are given. Number of observations: 455.

**shade level**	**emmean**	**SE**	**df**	**lCI**	**uCI**
with fixed effect year					
Shade < 20 %	1.52	0.22	450	1.09	1.96
Shade between 20 - 80 %	2.01	0.23	450	1.56	2.46
Shade > 80 %	3.19	0.34	450	2.52	3.86
without fixed effect year					
Shade < 20 %	1.52	0.23	452	1.08	1.96
Shade between 20 - 80 %	2.04	0.23	452	1.58	2.50
Shade > 80 %	3.20	0.35	452	2.52	3.88

**contrast**	**estimate**	**SE**	**df**	**lCI**	**uCI**	**t-ratio**	***P*-value**
with fixed effect year							
Shade < 20 % – shade between 20 - 80 %	0.49	0.32	450	-0.26	1.24	1.54	0.27
Shade < 20 % – shade > 80 %	-1.67	0.41	450	-2.63	-0.72	-4.12	0.00
Shade between 20 - 80 % – shade > 80 %	-1.18	0.41	450	-2.15	-0.22	-2.88	0.01
without fixed effect year							
Shade < 20 % – shade between 20 - 80 %	0.517	0.324	452	-0.24	1.28	1.60	0.25
Shade < 20 % – shade > 80 %	-1.68	0.41	452	-2.65	-0.70	-4.05	0.00
Shade between 20 - 80 % – shade > 80 %	-1.16	0.42	452	-2.14	-0.18	-2.77	0.02

emmean = estimated marginal mean
SE = standard error
df = degrees of freedom
lCI = lower 95 % confidence interval
uCI = upper 95 % confidence interval
