# Supplementary material for: Influence of light availability and soil productivity on insect herbivory on bilberry (Vaccinium myrtillus L.) leaves following mammalian herbivory
Source: PLoS One. 2020 Mar 27;15(3):e0230509. doi: 10.1371/journal.pone.0230509 (PMC7100976; doi:10.1371/journal.pone.0230509)
Supplement: S3 Table — Parameter estimates, standard error, 95% confidence interval and P-values are presented for the intercept and each of the fixed effects in the shade model. Note that the model used a logit link function (the estimates are on a logit-scale, not the response scale) and that the response variable was transformed prior to analyses (see text in the manuscript). Therefore, also the back-transformed estimates (back-transformed from both logit transformation and response variable transformation) are presented for the intercept and each of the fixed effects (thus, these values are on the response scale). Parameter estimates and 95% confidence interval are also presented for the standard deviation of the random component and for the dispersion parameter. Number of observations: 455. (PDF) [file pone.0230509.s004.pdf]

Table 3. Parameter estimates for the shade model of variables affecting insect herbivory on bilberry leaves. Parameter estimates, standard error, 95 % confidence interval and *P*-values are presented for the intercept and each of the fixed effects in the shade model. Note that the model used a logit link function (the estimates are on a logit-scale, not the response scale) and that the response variable was transformed prior to analyses (see text in the manuscript). Therefore, also the back-transformed estimates (back-transformed from both logit transformation and response variable transformation) are presented for the intercept and each of the fixed effects (thus, these values are on the response scale). Parameter estimates and 95 % confidence interval are also presented for the standard deviation of the random component and for the dispersion parameter. Number of observations: 455.

**Parameter**	**Estimate**	**SE**	**lCI**	**uCI**	***P*-value**	**Sign**	**BE**
Intercept	-3.89	0.10	-4.08	-3.70	0.00	***	0.02
Shade < 20 %	-0.23	0.09	-0.41	-0.06	0.01	**	0.44
Shade > 80 %	0.10	0.11	-0.13	0.32	0.40		0.52
Year 2014	0.38	0.09	0.20	0.57	0.00	***	0.60
Year 2015	-0.03	0.10	-0.22	0.15	0.73		0.49
Location (st.dev.)	0.15		0.05	0.42			
Dispersion parameter	47.70		40.49	56.15			

SE = standard error; lCI = lower 95 % confidence interval; uCI = upper 95 % confidence interval;
Sign = significance level: *** 0 < P < 0.001; ** 0.001 < P < 0.01; * 0.01 < P < 0.05; blank P > 0.05; BE = back-transformed estimates.
