# Supplementary material for: Influence of light availability and soil productivity on insect herbivory on bilberry (Vaccinium myrtillus L.) leaves following mammalian herbivory
Source: PLoS One. 2020 Mar 27;15(3):e0230509. doi: 10.1371/journal.pone.0230509 (PMC7100976; doi:10.1371/journal.pone.0230509)
Supplement: S4 Table — Model inferences based on Generalised Linear Mixed Modeling (beta regression with logit link). Best models based on AIC selection; only the five best models are presented, and the null model for comparison purposes. In addition to the presented model sets in the table, all models contained the random component (1|survey location). The first model (Δ AIC = 0.00) is the most parimonious model. The second model (Δ AIC = 0.50) is the full model. See text for description of the fixed effects and random component. Number of observations: 455. (PDF) [file pone.0230509.s005.pdf]

Table 4. Modeling of variables affecting insect herbivory on bilberry leaves in southeastern Norway in 2013-2015. Model inferences based on Generalised Linear Mixed Modeling (beta regression with logit link). Best models based on AIC selection; only the five best models are presented, and the null model for comparison purposes. In addition to the presented model sets in the table, all models contained the random component (1|survey location). The first model (Δ AIC = 0.00) is the most parimonious model. The second model (Δ AIC = 0.50) is the full model. See text for description of the fixed effects and random component. Number of observations: 455.

**Model set**	**df**	**AIC**	**Δ AIC**	**AICw**
Soil + Shade + Mammal + Year + Soil:Mammal + Shade:Mammal + Soil:Shade	14	-2642.33	0.00	0.49
Soil + Shade + Mammal + Year + Soil:Mammal + Shade:Mammal + Soil:Shade + Soil:Mammal:Shade	16	-2641.84	0.50	0.38
Soil + Shade + Mammal + Year + Soil:Mammal + Shade:Mammal	12	-2639.78	2.56	0.14
Soil + Shade + Mammal + Year + Shade:Mammal + Soil:Shade	13	-2627.44	14.89	0.00
Soil + Shade + Mammal + Year + Soil:Shade	10	-2627.12	15.22	0.00
Null	3	-2575.64	66.87	0.00

df = degrees of freedom; AIC = Akaike's information criterion; AICw = AICweight. Mammal = mammalian herbivory.
