# Supplementary material for: Influence of light availability and soil productivity on insect herbivory on bilberry (Vaccinium myrtillus L.) leaves following mammalian herbivory
Source: PLoS One. 2020 Mar 27;15(3):e0230509. doi: 10.1371/journal.pone.0230509 (PMC7100976; doi:10.1371/journal.pone.0230509)
Supplement: Attachment — Submitted filename: Response to Reviewers - MSG20200126.docx [file pone.0230509.s006.docx]

PONE-D-19-24380

Influence of light availability and soil productivity on insect herbivory on bilberry (*Vaccinium myrtillus* L.) leaves following mammalian herbivory

PLOS ONE

**Response to Reviewers**

Dear Academic Editor,

In this rebuttal letter, I respond to each point raised in the review process. First, I repeat the issue/point raised. My response follows after 'Response MSG:', see below.

One of our co-authors, Olivier Devineau, decided to withdraw as a co-author. This had been adjusted in the revised manuscript. In case you would like to have this confirmed by Olivier himself, he can be reached at: olivier.devineau@inn.no

With regards,

Marcel Schrijvers-Gonlag

Campus Evenstad, Faculty of Applied Ecology, Agricultural Sciences and Biotechnology

Inland Norway University of Applied Sciences, Koppang, Norway

marcel.schrijversgonlag@inn.no

**Journal Requirements:**

1. When submitting your revision, we need you to address these additional requirements.

Please ensure that your manuscript meets PLOS ONE's style requirements, including those for file naming. The PLOS ONE style templates can be found at
<http://www.journals.plos.org/plosone/s/file?id=wjVg/PLOSOne_formatting_sample_main_body.pdf> and <http://www.journals.plos.org/plosone/s/file?id=ba62/PLOSOne_formatting_sample_title_authors_affiliations.pdf>

Response MSG: I have studied the style templates in detail and have used these style requirements in the manuscript.

2. In your Methods section, please provide additional information regarding the permits you obtained for the work. Please ensure you have included the full name of the authority that approved the field site access and, if no permits were required, a brief statement explaining why.

Response MSG: I added this sentence: No permits for field site access were necessary, according to Norwegian law (friluftsloven: LOV-1957-06-28-16) that permitted access by foot to natural areas.

3. I am very sorry to hear that Harry P. Andreassen has passed away. Could you please confirm if there is any family member or next of kin we should contact if the manuscript is accepted for publication?

Response MSG: Not necessary to contact any relative of Harry, thanks for the offer.

**Comments to the Author**

1. Is the manuscript technically sound, and do the data support the conclusions?

The manuscript must describe a technically sound piece of scientific research with data that supports the conclusions. Experiments must have been conducted rigorously, with appropriate controls, replication, and sample sizes. The conclusions must be drawn appropriately based on the data presented.

Reviewer #1: Yes

Reviewer #2: Yes

Response MSG: no comment.

2. Has the statistical analysis been performed appropriately and rigorously?

Reviewer #1: No

Reviewer #2: Yes

Response MSG: I assume the reason for this 'no' (reviewer #1) is explained under '5. Review Comments to the Author', see my response there.

3. Have the authors made all data underlying the findings in their manuscript fully available?

The PLOS Data policy requires authors to make all data underlying the findings described in their manuscript fully available without restriction, with rare exception (please refer to the Data Availability Statement in the manuscript PDF file). The data should be provided as part of the manuscript or its supporting information, or deposited to a public repository. For example, in addition to summary statistics, the data points behind means, medians and variance measures should be available. If there are restrictions on publicly sharing data—e.g. participant privacy or use of data from a third party—those must be specified.

Reviewer #1: No

Reviewer #2: Yes

Response MSG: All data underlying the findings in the manuscript are fully and freely available via the following URL after the manuscript has been accepted for publication:

<https://doi.org/10.18710/89MLBP>

Before publication, this information can be accessed via a private URL:

<https://dataverse.no/privateurl.xhtml?token=87b6389f-b5cd-4453-9684-36b5cc1cc3f5>

4. Is the manuscript presented in an intelligible fashion and written in standard English?

PLOS ONE does not copyedit accepted manuscripts, so the language in submitted articles must be clear, correct, and unambiguous. Any typographical or grammatical errors should be corrected at revision, so please note any specific errors here.

Reviewer #1: Yes

Reviewer #2: Yes

Response MSG: no comment.

**5. Review Comments to the Author**

**Reviewer #1**: A review of a manuscript entitled “Influence of light availability and soil productivity on insect herbivory on bilberry (Vaccinium myrtillus L.) leaves following mammalian herbivory” for PLoS ONE (PONE-D-19-24380)

**General comments**
In this study, the authors aimed to evaluate whether the palatability of bilberry leaves (Vaccinium myrtillus) to insect herbivores is influenced by light availability, by soil productivity and by previous herbivory by mammals. A set of generalized linear models were used to test these three variables using a robust sample design. However, the results and discussion are dense, with many figures to look at and re-analyzes being described in the results and discussion. All of this part of data re-analysis to find the best model is part of the methods and I think it should not appear in the results. By reading the methods, the reader expects to see such analyzes and imagines such figures, but during the results the reader finds new analyzes being made based on the previous results. That does not seem appropriate to me here. These should all be described in the methods (or in supplementary documents) and the main results should be presented.

Response MSG: I have moved all the mentioned text parts to the Methods-section or to Supplementary documents, as suggested below by Reviewer #1.

This brings me to another issue. besides the text being dense in number of results, there are too many figures (14). In my opinion some tables and figures could be added as supplementary documents (e.g. Table 1, Figures 2, 3, 6, 8, and some ‘re-analyzed’ figures).

Response MSG: I have moved several text parts, tables and figures to supplementary documents. To improve readability, I have changed the method, result and discussion section extensively and unneccessary analyses (not necessary for the research question and the predictions) have been removed from the manuscript.

This ‘cleaning’ of the paper (i.e. reduction in the amount of results by focusing on the main ones) are my main suggestions, since the study deals with an interesting topic and the results are promising.

Response MSG: This 'cleaning' has been done carefully and extensively, see the revised version of the manuscript.

Minor suggestions are outlined below.

**Specific comments (L: lines)**

L 73-75: I suggest not leaving a sentence “alone” in the text. Please, add it to a paragraph.

Response MSG: This sentence is about the Growth Rate hypothesis. As we do not use this hypothesis in the manuscript, nor refer to it in the manuscript, I have deleted this sentence.

L 189-190: What is the criterion used to establish these categories? Distribution of data? There is a much greater amplitude in the intermediate category than in the others.

Response MSG: We expected the high and low values to have most impact, and in addition they may suffer least from sampling errors, so we made the very low and very high categories narrow. This is now explained in Methods.

L 474: Beware, the package itself shows nothing.

Response MSG: I have changed the sentence 'The DHARMa package showed ...' into 'Analyses with the DHARMa package showed ...'. I have done the same at line 684 (line number as in the first submitted version).

I could not access the data via DOI.

Response MSG: All data underlying the findings in the manuscript are fully and freely available via the following URL after the manuscript has been accepted for publication:

<https://doi.org/10.18710/89MLBP>

Before publication, this information can be accessed via a private URL: <https://dataverse.no/privateurl.xhtml?token=87b6389f-b5cd-4453-9684-36b5cc1cc3f5>

**Reviewer #2**: - The paper claims that bilberry palability by insects following mammalian herbivory is affected by leaf nutrient concentration and that this response is intensified by shade levels.
- The claims are properly placed in the context of previous literature but the readability of the introduction can be improved. Please see comments below.
- The analyses performed support their claims. However, figure 8 regarding the correlation between insect and mammalian herbivory must be improved. The analyses show a significant correlation but for some reason that’s not clear when looking at the figure.

Response MSG: The readability of the whole manuscript has been improved by removing less important information, analyses and figures. Figure 8, where Reviewer #2 refers to, has been moved to the Supplementary documents in our revised manuscript, as suggested by Reviewer #1. The direct correlation between insect and mammalian herbivory, as shown in this figure, is weak, but when soil productivity and shade conditions are taken into account the correlation becomes larger (described in the manuscript).

- Information on protocols and analyses seems to be complete.
- The paper could be published provided that the authors improve some aspects (please see comments below).
- Raw data is not included, it would be useful to include it.

Response MSG: All the data, including the scripts that we have used to obtain the results in the manuscript, are fully and freely available via the following URL after the manuscript has been accepted for publication:

<https://doi.org/10.18710/89MLBP>

Before publication, this information can be accessed via a private URL:

<https://dataverse.no/privateurl.xhtml?token=87b6389f-b5cd-4453-9684-36b5cc1cc3f5>

- Details of the methodology are sufficient.
- Yes, the manuscript is well organized and written clearly.


**Comments**
Line 54: There is no connection between the previous paragraph and the paragraph starting in this line. There is a phrase missing introducing this new hypothesis. How are connected the constitutive defense and the optimal defense (OD).

Line 60: Similarly as above, the carbon:nutrient balance (CNB) hypothesis comes out of the blue. Could you please introduce with an explanatory phrase on how this hypothesis is connected with the previous one?

Response MSG: I added two connecting sentences before introducing the three defense hypotheses to improve readability.

Line 140 to 152: Probably it would be easier for the reader if you integrate this information in the text of the introduction to connect your own hypothesis with the one in the literature.

Response MSG: The sentences that I have added (see my response to the comment above) connect the provided theoretical information to our own predictions.

Line 218: Which are the productivity classes? They have not been introduced so far.

Response MSG: The soil productivity classes have been introduced in lines 214-217.

Figure 4a: The scales are so different between insect herbivory and mammalian herbivory that it is difficult to see the trend of the effect of shade on mammalian herbivory. Please separate these two figures.

Response MSG: Figure 4 has been changed (is now Figure 3) and this figure has only one axis now. The figure with mammalian herbivory has been removed.

Why in the statistics of figure 4 (lines 303 to 310) there is a report of F and P values of years 2013 and 2014? What about year 2015? Please include missing information.

Response MSG: In the revised manuscript, we removed the analyses on separate years.

Why mammals feeding on low soil productivity levels?.

Response MSG: In the revised manuscript, we removed the analyses on mammalian herbivory and soil productivity (not part of our research question nor our predictions and this improves readability of the manuscript).

Also, there was something going on in 2013: -High mammalian herbivory in quadrats with high shade levels than at quadrats with low shade levels. - there was no difference in mammalian herbivory between the three soil productivity classes. While for the other years a higher mammalian herbivory was registered for low soil productivity. Could you comment on that ?

Response MSG: In the revised manuscript, we removed the analyses on mammalian herbivory and shade as well as the analyses on mammalian herbivory and soil productivity (both are not part of our research question nor our predictions and this improves readability of the manuscript).

Figure 6. Please explain briefly in the figure title what you mean with “standardized mammalian herbivory”.

Response MSG: The “standardized mammalian herbivory” is mentioned in the main text (line 223-224). Figure 6 has been deleted in the revised manuscript.

Figure 8. Why an R2 value is not presented in the figure. Please include it. Can you please explain in the figure legend what you mean by “adjusted” R2.

Response MSG: The R^2^-value is mentioned in the figure legend. This whole figure 8 has been moved to Supplementary documents. We present the adjusted R^2^ instead of the non-adjusted R^2^ as the adjusted R^2^ is dependent on the number of variables in the model and adjusts for sample size.

The correlation does not seem very strong when the shade variable is taken away…Especially taking into account that the Y axis goes only 35%. It should be 100%.

Response MSG: I assume that this comment refers to Figure 9 in the submitted manuscript (becomes Figure 5 in the revised manuscript). Yes the variable shade is very important in the predicted results and this is mentioned in our results (lines 320-325) and our conclusions (lines 427-432). The y-axis is deliberately presented up till 35 % as the predicted values have maximum values around the value 30. By presenting values only up to 35% at the y-axis the lines in the panels are easier to distinguish from each other.
